# Monitoring the newly qualified nurses in Sweden: the Longitudinal Analysis of Nursing Education (LANE) study

**DOI:** 10.1186/1478-4491-8-10

**Published:** 2010-04-27

**Authors:** Ann Rudman, Marianne Omne-Pontén, Lars Wallin, Petter J Gustavsson

**Affiliations:** 1 Division of Psychology, Department of Clinical Neuroscience, Karolinska Institutet, SE-17177 Stockholm, Sweden; 2 Division of Nursing, Department of Neurobiology, Care Sciences and Society, Karolinska Institutet, SE-14183 Huddinge, Sweden

## Abstract

**Background:**

The Longitudinal Analysis of Nursing Education (LANE) study was initiated in 2002, with the aim of longitudinally examining a wide variety of individual and work-related variables related to psychological and physical health, as well as rates of employee and occupational turnover, and professional development among nursing students in the process of becoming registered nurses and entering working life. The aim of this paper is to present the LANE study, to estimate representativeness and analyse response rates over time, and also to describe common career pathways and life transitions during the first years of working life.

**Methods:**

Three Swedish national cohorts of nursing students on university degree programmes were recruited to constitute the cohorts. Of 6138 students who were eligible for participation, a total of 4316 consented to participate and responded at baseline (response rate 70%). The cohorts will be followed prospectively for at least three years of their working life.

**Results:**

Sociodemographic data in the cohorts were found to be close to population data, as point estimates only differed by 0-3% from population values. Response rates were found to decline somewhat across time, and this decrease was present in all analysed subgroups. During the first year after graduation, nearly all participants had qualified as nurses and had later also held nursing positions. The most common reason for not working was due to maternity leave. About 10% of the cohorts who graduated in 2002 and 2004 intended to leave the profession one year after graduating, and among those who graduated in 2006 the figure was almost twice as high. Intention to leave the profession was more common among young nurses. In the cohort who graduated in 2002, nearly every fifth registered nurse continued to further higher educational training within the health professions. Moreover, in this cohort, about 2% of the participants had left the nursing profession five years after graduating.

**Conclusion:**

Both high response rates and professional retention imply a potential for a thorough analysis of professional practice and occupational health.

## Background

### Nurse shortage

The main current problem for healthcare organizations worldwide is the shortage of health service providers [1]. This shortage is due to the increasing consumption of healthcare and a growing population that lives longer, in combination with an ageing nursing workforce, migration, reduced working hours, early retirement and the tendency of nurses to leave the profession [2-7]. Other problematic issues involve attrition from undergraduate programmes and retention of recent graduates within the workforce [8].

### Occupational turnover: giving up the nursing profession

Nurses' health, working conditions, job satisfaction and occupational commitment affect nurse behaviour such as turnover, which in turn can influence quality of care and patient outcomes [9-13]. As a result, nurse turnover has been a growing subject of interest. Unfortunately, the research base is largely inconsistent in definitions used [5,6]. Hayes and coworkers highlight this in their review of nurse turnover literature: "Some studies define turnover as any job move, while others consider nurse turnover as leaving the organization or even the nursing profession" p. 238-9 [5]. Therefore, despite the significance of turnover, it is challenging to interpret and compare across different studies, healthcare systems and countries [5,6].

The multinational European Nurses Early Exit (NEXT) study showed that intention to leave the nursing profession (occupational turnover) provided a good estimate of subsequent decisions to actually quit [14]. Hayes and co-workers also found that intention to leave was positively related to actually leaving [5]. In 2002, almost 16% of European nurses frequently considered leaving the nursing profession. When divided by country, 32% in the United Kingdom of Great Britain and Northern Ireland often considered leaving; the corresponding number in Italy was around 21%, while less than 10% of nurses in the Netherlands and Belgium reported that they intended to quit. In the participating Scandinavian countries, Norway and Finland, the proportions were 12% and 14% respectively. At follow-up in the NEXT study, a total of 9.3% of nurses had in fact left the nursing profession (ranging from 4.5% in Italy to 14.6% in Germany) [14]. In Canada, 13% of young, newly qualified nurses (who received their professional qualifications during 2004) intended to leave the profession [15]. The United States RN workforce may eventually shrink, owing to a decline in the number of younger women who choose a career in nursing [16]. Problems of attrition from undergraduate programmes and retention of recent graduates within the workforce were also reported in Australia [8]. Similarly, in the United Kingdom, approximately one in seven newly qualified nurses and midwives chose not to enter their profession at all [17]. However, considering that the shortage of nurses is a worldwide problem, there is a striking lack of research that systematically and longitudinally investigates attrition and retention, as well as reasons why new graduates leave the profession [8].

In 2006, 12% of the nursing workforce in Sweden were not employed within the healthcare system [18]. Nurses who left the profession entirely have given multiple reasons, e.g. legal and employer issues, stressful or poor working conditions, working life/home life and effort/reward imbalances, as well as external values and beliefs about nursing (e.g. low status of profession) [19-21].

### Nurses' health

Numerous studies are either directed towards an investigation of student health outcomes [22], or working conditions and health after entering working life [23,24]. During the last decade in Sweden, some professional groups, including nurses, have been affected by an increasing frequency of stress [25,26] and long-term sick leave [27]. An increasing prevalence of mental ill health has been regarded as the primary explanation behind these figures. Research into stress and professional health has shown that quality of care was compromised due to processes of burnout, and that in time staff accomplished less and became more exhausted and disengaged [28]. The connection between nurse burnout and concerns about quality of care was supported by the work of Aiken and colleagues [10,29]. They found that patients who were cared for at units where nurses reported significantly lower burnout were more likely than other patients to report high satisfaction with their care. Other studies identified that job stress and burnout was related to turnover and intention to leave the profession [5,6,21].

### Swedish labour market and nursing education

In Sweden, the labour market demand for nurses is relatively good, with an unemployment rate of below 0.5% [30]. The density of nursing and midwifery personnel in 2002 was approximately 100 per 10 000 inhabitants, which is relatively high compared with the rest of the world (http://www.who.int/whosis/en/index.html (accessed 5 March 2009)). While the labour market is somewhat balanced in supply and demand, the nursing educational system has undergone major structural changes. One educational change that has occurred during the past 15 years is the transition from a non-academic and practically oriented education programme to higher education leading to an academic degree [31,32]. Concurrently, the number of students on these higher education programmes has increased. For instance, the number of places within Swedish nursing programmes (full-time equivalents) expanded from 3000 to 4500 places between the years 2000 and 2005 [33]. Since the year 2000, the number of nurses (of working age) has therefore grown and there are now over 130 000 nurses in Sweden. However, the increase in the number of places on the nursing programmes has also led to a related decrease in minimum entry qualifications, problems in recruiting senior lecturers, and a struggle to establish an adequate level of clinical training. Consequently, learning conditions have differed from one study centre to another within Sweden, with respect to number of students per teacher, senior competence, availability of well functioning clinical practice and students' preparedness for higher educational studies [34].

Thus, the future retention and continuing professional development of nurses, and the provision of high quality care, may depend on the interaction of many factors, such as student engagement and commitment, outcome of higher educational studies, working conditions and occupational health. In order to address several of these issues, a nationwide study (the Longitudinal Analysis of Nursing Education, LANE) was initiated in 2002, with the aim of longitudinally examining a wide variety of individual and work-related variables related to psychological and physical ill health and well-being, as well as risk and protective factors for mental ill health among nursing students in the process of becoming registered nurses and entering working life. The database, now under construction, will make it possible to study individual conditions, educational structures, and contextual factors in healthcare that affect health trends among new graduates in the transition from undergraduate studies to practice.

Thus, the aim of the LANE study is to monitor the health status as well as retention, turnover rates (for both employee and occupational turnover) and professional development of newly qualified nurses in their first years of working life. The aims of this paper are to present the LANE study, to estimate representativeness and analyse response rates over time, and also to describe common career pathways (including intention to leave nursing and occupational turnover) and life transitions during the first years of working life.

## Methods

### Sampling frame

In Sweden there are approximately 130 000 registered nurses (under 65 years of age), and another 40 000 new graduates will enter the labour market during this first decade of the century. In all, there are about 300 000 students in higher education and around every 20^th ^student is taking an undergraduate nursing programme (with the goal of becoming a registered nurse with a bachelor's degree). The three cohorts that comprise the LANE study include nursing students who were expected to graduate and receive their nursing degree in the autumn of 2002, 2004, and 2006, respectively. In the following text, these three cohorts are referred to as the EX2002, EX2004, and EX2006 cohorts. Participants eligible for the study were 6138 nursing students attending a predefined semester at any of the 26 Swedish universities offering nursing programmes in Sweden at that time (see Table 1). Lists of students were taken from the national registry of educational statistics, comprising all students taking a higher educational programme or course in Sweden [35]. For the EX2002 and EX2004 cohorts, lists were administered and collected separately from each university. Two universities did not consent to provide the research group with these lists. At these two universities, the students were informed about the study by university staff, who asked for permission to pass on their names and addresses to the research group. Unfortunately neither the exact number of students attending these universities nor the number of students who were informed about the study is known to the research group. But based on official figures of examination rates for these two universities, the proportion of students eligible and listed in the sampling frame should comprise at least 75% of all students in the EX2002 cohort, but not more than 50% in EX2004. For EX2006, the list was provided directly from the national register by Statistics Sweden (the central government authority for official statistics and other government statistics in Sweden).

**Table 1:** Distributions of age and sex among eligible students and students who consented to participate.

			EX2002	EX2004	EX2006
**Sampling frames**	**N**	Number of eligible students	1700	2331	2107
		
		Semester when first assessed	6^th ^out of 6	2^nd ^out of 6	6^th ^out of 6
		
		Expected graduation	end of 2002	end of 2004	end of 2006
	
	**Sex**	% of females	88.2	87.9	86.0
		
		% of males	11.8	12.1	14.0
	
	**Age**	Mean	30.5	28.3	29.8
		
		Standard deviation	7.4	7.2	7.1
		
		Range	21 - 54	20 - 57	21 - 55
		
		% aged ≤24	27.5	41.9	29.2
		
		% aged 25 - 34	43.2	36.3	46.2
		
		% aged ≥35	29.3	21.8	24.6

**Cohorts**	**N**	Number in cohort	1155	1702	1459
	
	**%**	Response rate	67.9	73.0	69.2
	
	**Sex**	% of females	89.2	89.1	89.0
		
		% of males	10.8	10.9	11.0
	
	**Age**	Mean	30.5	28.4	29.9
		
		Standard deviation	7.4	7.2	7.1
		
		Range	21 - 52	20 - 52	21 - 54
		
		% ≤24	28.4	42.0	28.9
		
		% 25 - 34	42.1	36.1	46.2
		
		% ≤35	29.5	21.9	24.9
	
	**Rate**	Highest response rate to date	91.7	92.1	78.1
		
		Lowest response rate to date	80.8	69.0	78.1

Note: Administrative data taken from the sampling frames.

Students who were eligible for participation in EX2002 and EX2004 were informed about the study, either by attending an oral presentation given by a member of the research group at their university, and/or by an information letter sent to each student. At the presentation seminar, written information about the study and the survey instrument was available. Those who did not attend the information meeting, or for whom there was no record that they had received the written information at the seminar, were contacted by post. All EX2006 students were contacted by post. After two reminders (the last one including a new information letter and a copy of the questionnaire), the students who gave their consent (and thus constituted the cohorts) were defined. Since 2003, all data collections have been administered by Statistics Sweden. Data from the sampling frames for the LANE study are presented in Table 1.

### Study design

The study has an observational longitudinal design [36], where the development of individual health outcomes, professional competence and patterns of employment, intention to leave the nursing profession and early retention in the workforce will be investigated. Annual data collection started in 2002 (for the EX2002 and EX2004 cohorts, but in 2006 for the EX2006 cohort) and will continue until 2010. As the focus in this study is on the transition from higher education into working life, the three cohorts will all be annually measured on at least four occasions, as the observational period extends from the last semester of nursing education up to 3 years after graduation. In addition, supplementary measurement will be performed at specific time points in two of the cohorts. The EX2002 cohort will be followed-up five years after their graduation, i.e. five measurement occasions in all. For the EX2004 cohort there are two additional measurement occasions during their nursing education (in the second and fourth semester), as well as two additional measurements four and five years after their graduation (a total of eight measurement occasions). In this present paper, data from baseline as well as from the first year after graduation will be presented for all three cohorts. In addition, data from all measurement occasions will be presented for the first cohort, where data collection has been completed (i.e., EX2002).

The objectives for choosing a multiple-cohort observational design were: to measure and compare period and cohort effects, and to describe developmental change and temporal order of events in relation to health status and incidence of disease [37]. The word cohort here refers to "a group of people who share a common experience or condition" p. 79 [38], in this case pursuing nursing education and entering the nursing labour force. The design chosen was advantageous here because many different risk factors were of interest during studies, in the changeover from studies to practice, and after a period within the workforce [39]. Also, by inviting nurses to participate while they were still students, baseline assessments could be used to adjust for and take into account the potential influence of individual and educational factors on outcomes after entry to working life.

The following steps were taken to ensure an appropriate sample size that could give stable and correct estimates of main outcomes such as depression and later job burnout. The argument focuses on changes in mean levels of depression over time in an affected group. The strategy here was to find the power to perform a post-hoc test, where the increase in mean levels of depression could be detected in an affected group and compared with a non-affected group. To detect a small mean change in symptom levels (i.e. an effect size of 0.20 standard deviations) across two adjacent time points with a power of 80%, 199 affected subjects are required [40].

According to data from community surveys addressing point estimates of depressive symptoms, up to 20% of the adult population are affected [41]. Moreover, as the prevalence has been found to be higher for females [42] and for younger adults [41], this certainly implies that 20% may be a rather low estimate of symptom prevalence in the present population of students. Extrapolating from these data, it is necessary to include 199 subjects times five (i.e. about 1000 subjects), resulting in a power of 81%, to detect a small mean level difference between an affected and a non-affected group. Given that the population of eligible nursing students during years 2001-2006 ranged between 1700 and 2300, it was decided to include all students from a defined semester, in order to guarantee a large enough sample from the outset. Accounting for the fact that about 30% decline to participate, this amounts to an actual minimum of 1190 students, resulting in a power of at least 87%, to detect both a small mean level change across time and a small group difference.

Approval for the initial study consisting of the two first cohorts was received from the regional Research and Ethics Committee at Karolinska Institutet, Sweden (Dnr 2005/1532-32). Additional permission regarding another cohort (EX2006) and subsequent data collections and questionnaires was received (Dnr: KI 01-045 [2001-05-14; 2003-02-29]; 04-587 [2004-08-08]; 05/321-32 [2005-0323]; 06/973-32 [2006-08-29] 2008/226-32 [2008-02-12]). Written informed consent was obtained from all study participants. To minimize the risk of ambiguity or distress, oral and written information was given, and a covering letter also accompanied each questionnaire. The covering letters kept the study participants updated and always included details of how to contact the research team. The research team was available to answer questions and concerns by phone and e-mail.

### Data

All data in the LANE study are self-reported and collected by means of a postal survey, except for year of birth, sex and social security number, which were originally retrieved from the national registry of educational statistics and later validated by comparisons with data given by participants in their written informed consent. Also, to ensure quality over time, each survey was reviewed by the workers at the technical and language laboratory at Statistics Sweden (SCB). General background variables included civil status, household composition, previous education, social support and critical life events and were asked in each wave of data collection when appropriate. Most main outcomes were assessed repeatedly in all cohorts, but EX2002 had a unique focus on occupational values; EX2004 was specifically oriented towards a complete coverage of assessments related to education, personality factors and research utilization; and EX2006 had an extended focus on psychosocial factors at work, and was suitable for comparison with another project on teaching students.

### Measurement

The main psychological health outcomes, i.e. depressive symptoms and job burnout, were measured by the Major Depression Inventory [43] and the Oldenburg Burnout Inventory [44], respectively. Additional health aspects included were self-rated health, sleep quality, dental health, height, weight, healthcare utilization, and self-reported prevalence and impact of musculoskeletal, allergy and eczema symptoms. Subjective well-being was measured by the Life Satisfaction scale [45]. Questions related to health behaviours were alcohol consumption, smoking and eating habits, as well as exercise and physical activity. Personality traits were assessed using the Health-relevant Personality traits from a five factor perspective - HP5 inventory [46], the Performance-based self-esteem scale [47], and Bandura's academic efficacy scales [48].

Professional competence and practice was conceptualized as occupational self-efficacy [49] and research utilization (RU) [50], and measured by items adapted from Bandura's self-efficacy scales [48] and Estabrook's RU measures [51]. Occupational variables comprised employment details, income, job history and reasons (and/or intentions) for leaving a position or the profession. Questions on work setting, nature and duration of shift work, ergonomic strain and sickness absence were also included. Furthermore, psychosocial work characteristics were assessed by scales using the Nordic Questionnaire of Psychosocial factors at work [52], including scales capturing job demands, control, mastery, role conflicts, as well as social support and leadership.

Items from the National Survey of Student Engagement [53] were used to assess graduate outcomes, including student engagement, quality and outcome of undergraduate training.

At the end of the 25-page questionnaire, two open-ended questions were added, where the respondents were invited to write comments on subjects of current importance to them. Initially, the open-ended questions primarily focused on encouraging participants to outline important areas that had not yet been covered in the questionnaires. Subsequently, the general question was phrased: "If you have any thoughts about yourself, or the LANE study, that you would like to share with us, and which have not been covered in the questionnaire, please write your comments below!" In addition, an open-ended question suitable for the specific time point was generally asked, covering areas such as: expectations of the nursing profession; experience of a) incongruity or agreement between the theoretical and practical part of the study programme; b) the introduction to establishing a professional nursing role in working life, c) factors important for the transition from education to practice, d) significant events related to nursing work.

Background variables, employee status, and items concerning life events and intention to leave the nursing profession will be analysed in the present paper.

### Data analysis

Cohort representativeness was evaluated using data from population-based national registers. Demographic characteristics of the total population of nursing students and the ones who consented to participate were compared. From the national register population, values of age, gender, country of birth, residency (large city), marital status, and parenthood, were defined for nursing students in their 6^th ^and final semester in the autumn of 2002, 2004, and 2006, respectively. Point estimates as well as confidence intervals for these data in the three cohorts were computed and compared with population values. As these data from the national registers are not available to the research group, these comparisons were performed by Statistics Sweden.

A longitudinal analysis of response rate was performed (where response or non-response was measured at every follow-up assessment). Because so few data collections have been conducted on the EX2006 cohort up to this point, analyses were only performed on the EX2002 and EX2004 cohorts. Factors influencing participants' response rates across time were evaluated using self-reported data from the baseline questionnaires as predictors of participation. Age, gender, country of birth, civil status (cohabiting or not), as well as self-rated health were used as time-invariant predictors of the longitudinal change in participation rates. Data were analysed using a regression procedure referred to as 'longitudinal logistic regression' [36], 'marginal logistic regression' [54] or 'repeated measures logistic regression' [55], using Generalized Estimation Equations in PASW Statistics 18 [55]. The main effects of time, as well as the interaction of each predictor with time, were tested with the Wald Chi-square statistic. The effects were further described by plotting the estimated response rates for all predictors by time interactions and by the computation of post-hoc tests (of the simple effects).

Both the robust and the model-based estimators were tried in combination with different structures of the working correlation matrix (AR[1], Exchangeable, M-dependent, and Unstructured). These different tests are not presented, as they yielded almost identical results. The results shown here are based on the model-based estimator and an unstructured working correlation matrix.

## Results

### Recruitment and retention

Of 6138 students who were eligible for participation, a total of 4316 consented to participate (a participation rate of 70%). Furthermore, of the 4316 that consented to participate, 10 (0.2%) did not subsequently complete the questionnaire at baseline. Response rates across the three cohorts varied between 68 and 73%, giving the highest response rate in the cohort recruited earlier in their education. Administrative data for eligible students from the sampling frame and consenting students are presented in Table 1. For all three cohorts, age distributions are close to data presented for the sampling frame. However, the percentage of participating females is about 89% in all three cohorts, which is 1-3% higher than in the sampling frame.

Cohort representativeness was further evaluated by comparing point estimates as well as confidence intervals for consenting nursing students in their sixth and final semester in the autumn of 2002, 2004, and 2006 against national register population values on six different demographic variables among all nursing students registered for the sixth semester in those particular years (for a demographic description of the cohorts at baseline, see Tables 1 and 2). The absolute difference between population prevalence and cohort estimates ranged from 0 to 3 per cent (mean 1.2%), and the confidence intervals (95%) included the population values in 14 out of 18 comparisons. For EX2004, no significant population and cohort differences were found. The EX2002 cohort differed from the population in one instance: the prevalence of Swedish-born students was 3% units higher than in the population. The EX2006 cohort differed from the population in three instances: in this cohort, both the prevalence of female participants and Swedish-born students was 2% higher than in the population, whereas the prevalence of students living in large cities was 2% lower than in the population.

**Figure 1:**
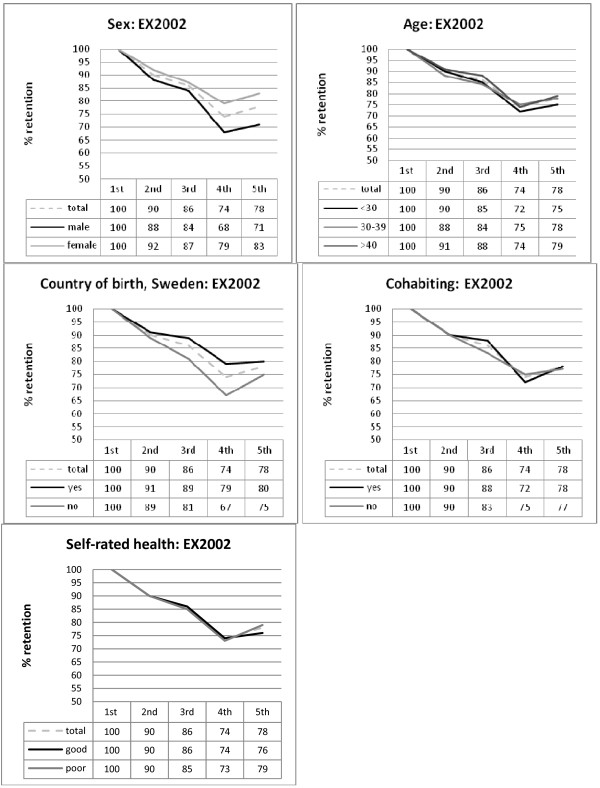
**Estimated annual response rates (adjusted means) to postal questionnaires in the LANE EX2002 cohort, with respect to sex, age, country of birth, cohabiting and self-rated health**. Note: Estimates taken from the repeated measures logistic regression analysis.

**Figure 2:**
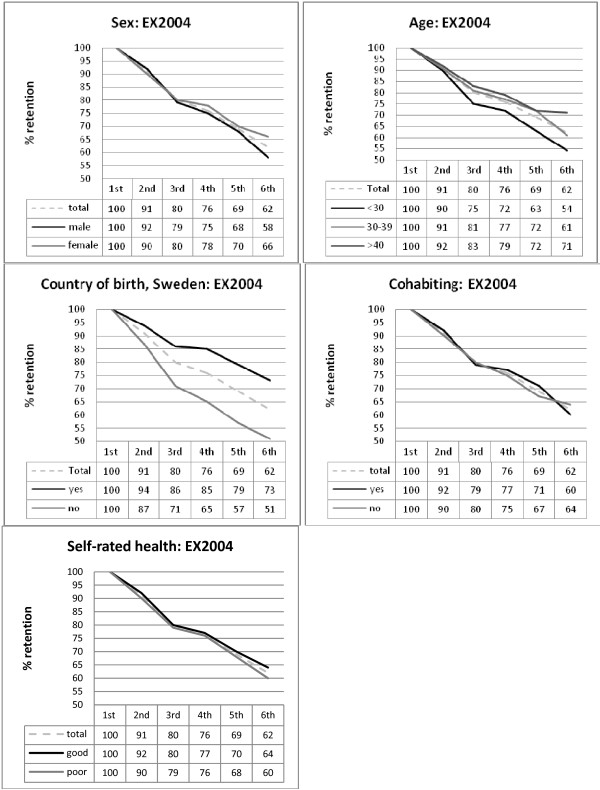
**Estimated annual response rates (adjusted means) to postal questionnaires in the LANE EX2004 cohort, with respect to sex, age, country of birth, cohabiting and self-rated health**. Note: Estimates taken from the repeated measures logistic regression analysis.

The possible influence of demographic factors on changes in response rates across time was analysed, using a repeated measures logistic regression, estimated using Generalized Estimation Equations. The main effects of time, as well as the interaction of each predictor with time, were tested with the Wald Chi-square statistic, and are presented in Table 3. In both cohorts, the main effect of time reflects that the response rates at different measurement waves vary across time (actual response rates are given in Table 1 and adjusted response rates estimated from the regression analysis are given in Figures 1 and 2). Post-hoc analyses showed that there is a decline in response rates over time, and this decline is present in both the total cohorts and in every subgroup analysed (see Figures 1 and 2). That the decline in response rate follows a similar pattern in all subgroups is also reflected in that only one (out of ten) interaction effect (one effect for the X2004 cohort) was found to be statistically significant. However, an inspection of the estimated response rates for this interaction effect (cohabiting by time) reveals that the actual differences are small and the post-hoc analyses showed no significant differences between the groups on any measurement occasion.

**Table 2:** Background characteristics among students who consented to participate.

	EX2002	EX2004	EX2006
**Country of birth**			

Swedish: Yes	93.8	91.2	91.1

**Previous education**			

% Training as nursing assistant	42.5	45.4	35.8

% Higher education	22.1	25.1	28.9

% Bachelor's degree	2.6	2.9	6.3

**Previous work experience** **in the healthcare sector (%)**	54.1	60.1	54.1

**Nurse among relatives (%)**	42.2	44.0	44.2

**Civil status**			

% Cohabiting	66.5	62.0	63.6

% With children	43.0	40.1	39.0

**Self-rated health**			

% good	60.4	58.9	48.8

Note: Data taken from the baseline questionnaires.

**Table 3:** Analysis of response rate across time (for the EX2002 and EX2004 cohorts).

	EX2002		EX2004	
	**Wald χ**^**2**^	**sig.**	**Wald χ**^**2**^	**sig.**

**Constant**	97.50	0.001	137.40	0.001

**Time**	23.20	0.001	92.92	0.000

**Sex**	6.17	0.012	0.18	0.669

**Age**	0.74	0.687	10.26	0.005

**Swedish-born**	2.71	0.099	45.53	0.001

**Civil status (cohabiting)**	0.39	0.527	0.48	0.485

**Self-rated health (SRH)**	0.00	0.935	1.48	0.223

Time*sex	1.56	0.666	4.94	0.293

Time*age	3.20	0.782	7.58	0.474

Time*Swedish-born	1.86	0.600	1.53	0.819

Time*cohabiting	6.94	0.073	14.61	0.005

Time*srh	3.27	0.351	0.94	0.918

Data from a repeated measures logistic regression, predicting change in participation/response (vs non-participation) from time (measurement wave), sex, age, country of birth, civil status and self-rated health.

Furthermore, the significant main effect of gender on response rate in the EX2002 cohort suggests that response rates for the male subgroup are lower across time, but only statistically significant in the post-hoc analyses for the last two measurement waves. Similarly, the significant main effect of age on response rate in the EX2004 cohort suggests that response rates for the youngest subgroup are lower across time, and statistically significant for the last two measurement waves. In contrast, a significant main effect of country of birth on response rate in the EX2004 cohort suggests that response rates for the non-Swedish-born subgroup are lower across time, and statistically significant in the post-hoc analyses for all follow-up assessments.

### Baseline

Numbers of participants, as well as age and sex distributions in the three cohorts, are presented in Table 1. When comparing the percentage of answers between the EX2002, the EX2004 and the EX2006 cohorts (Table 1), the different recruitment methods probably did not affect uptake percentages as much as the difference in number of years spent in education at the time of recruitment. In other words, the higher response rate in cohort EX2004 most likely relates to the fact that they were recruited in the second semester as opposed to the sixth, which was the case for the other two cohorts.

The age distribution is similar in the two cohorts recruited during their final semester. Consequently, the mean age is about two years lower in the cohort recruited during their first year of nursing studies (i.e. EX2004). Table 2 shows demographic characteristics (originating from the baseline questionnaires) for students in all three cohorts. Although the three cohorts are quite similar along most variables, some small but notable differences might be of interest. As was already shown in the representative analyses above, fewer students in the EX2002 cohort were born in a country other than Sweden (6% vs. 9% in EX2004 and EX2006). Students in the EX2006 cohort have more often participated in previous higher education and obtained bachelor's degrees; at the same time, they less often have previous training as nursing assistants. In addition, they do not rate their health as highly as the students from the other cohorts do. Students in the EX2004 cohort have slightly more previous work experience from the healthcare sector, and more often have previous training as nursing assistants. Finally, with respect to civil status, students in the EX2002 cohort cohabit slightly more often than the other students.

### One year after graduation

The response rates in all three cohorts one year after graduation (corresponding to the second wave in the EX2002 and EX2006 cohorts, but the fourth wave in the EX2004 cohort), are presented in Table 4. Over 90% of the respondents in the EX2002 cohort participated in the assessment one year after graduation. The rate was somewhat lower (around 80%) in the other two cohorts.

**Table 4:** Experience of working life one year after graduating.

	EX2002	EX2004	EX2006
**Year of data collection**	2004	2006	2008

**Wave number**	2	4	2

**Response rate (%)**	91.7	82.3	78.1

**Have obtained nursing qualification (%)**	99.0	93.9	97.8

**Have worked as an RN since graduation (%)**	98.9	96.0	98.0

**Currently working as an RN (%)**	92.0	92.5	91.3

**Hold a permanent position as an RN (%)**	78.0	38.2	48.3

**Working full-time as an RN (%)**	78.4	68.8	74.4

Data on nursing qualifications and employment one year after graduation are also presented in Table 4. During the first year after graduation nearly 100% had received their nursing qualifications and almost all had worked as a registered nurse at some point since graduation. At the time of data collection, about 92% were currently working as registered nurses. The most common reason for not working was due to maternity leave. Of those presently working, almost 80% in the EX2002 cohort (but only 38% and 48% in the EX2004 and EX2006 cohorts, respectively) held permanent positions. In addition, 78% in the EX2002 cohort, 69% in the EX2004 cohort, and 74% in the EX2006 cohort worked as a nurse on a full-time basis.

Percentages of nurses intending to leave the profession one year after graduation are presented across the three cohorts in Table 5. In general, the percentages for the EX2002 and EX2004 cohorts are comparable, while the percentages in the EX2006 cohort are consistently higher. For example, the percentage of nurses with frequent thoughts about leaving the profession is about 10% in the EX2002 and EX2004 cohorts, and almost twice as high in the EX2006 cohort. Similarly, the percentages of nurses who actively seek positions outside the profession, or have a strong desire to leave the profession, are higher in the EX2006 cohort in comparison with the other two cohorts. No differences are found between the sexes on any of the three items concerning intention to leave the nursing profession. For the two intention items, reflecting active job-seeking and an immediate desire to leave, there are no differences between different age groups. However, in all three cohorts the youngest group shows consistently higher percentages of nurses who often think about leaving the profession.

**Table 5:** Intention to leave the nursing profession, presented as the percentage of newly qualified nurses who have had such intentions during their first year after graduation, in the three cohorts.

	Total (%)	Sex (%)		**X**^**2**^**(p)**	Age (%)			**X**^**2**^**(p)**
		**Male**	**Female**		**≤29**	**30 - 39**	**≥40**	

**Often think about leaving the profession (%)**								

**EX2002**	10.1	13.2	9.8	1.2(n.s)	14.8	7.5	4.0	22.7(.001)

**EX2004**	9.2	9.9	9.0	0.1(n.s)	11.8	8.9	4.0	12.3(.002)

**EX2006**	19.0	23.2	18.5	1.5(n.s)	24.5	13.5	13.1	21.8(.001)

**Actively applying for positions outside the profession (%)**								

**EX2002**	1.9	1.0	2.0	0.6(n.s)	2.3	1.6	1.4	0.9(n.s)

**EX2004**	1.5	2.5	1.3	1.0(n.s)	2.3	1.3	0.0	6.1(.047)

**EX2006**	3.0	4.5	2.9	0.8(n.s)	3.4	2.7	3.0	0.3(n.s)

**Have a strong desire to leave the profession immediately (%)**								

**EX2002**	2.0	1.9	2.0	0.18(n.s)	2.5	2.0	0.9	2.0(n.s)

**EX2004**	1.7	1.7	1.7	0.1(n.s)	1.9	1.0	2.4	2.0(n.s)

**EX2006**	3.0	4.5	2.8	0.9(n.s)	3.0	3.0	3.0	0.1(n.s)

### The first five years after graduation

Data collections for the EX2002 cohort were completed in 2008, more than five years after the former students had graduated. In this cohort the actual response rate varied between 81% and 92% across five years (c.f. 69% to 92% in EX2004). Only 38 respondents dropped out after the baseline assessment. Data reflecting retention and common life transitions across the years after graduation will be briefly summarized for this cohort below. Five years after graduation, at least 97% of all participants in the EX2002 cohort had worked as registered nurses at some time point. At this time, only a few persons had not completed their bachelor's degree. Moreover, every fifth nurse in the EX2002 cohort had returned to further higher educational training, and had graduated as a midwife, or gained a graduate diploma in specialist nursing. In addition, at least 2% of the participants in the EX2002 cohort had left the nursing profession. There are other common life events parallel to graduation and entering working life that are known to affect health. For example, data from the EX2002 cohort reveal that study participants give birth to approximately 100 babies each year up to three years after graduation. In addition, about 110 new marriages and 50 to 75 divorces were reported each year up to three years after graduation. Furthermore, 0.6% of the EX2002 cohort report a period of unemployment at some time during their first three years of working life.

## Discussion

In the ongoing LANE study, a 70% response rate was found at baseline, when the three cohorts were established. Subsequent actual participant rates across data collection waves were high, ranging between 69% and 92%. In the sixth semester the three cohorts were found to be representative of the populations, as point estimates of sociodemographic data were close to population data, only differing about 0-3% from population values. Response rates were found to decline somewhat across time (11% units in EX2002 and 23% units in EX2004), and this decrease was present in all analysed subgroups. Importantly, self-rated health was not associated with attrition. The most consistent demographic variables showing any influence on recruitment and response rates were gender (males being either underrepresented in one cohort by 2% units or showing declining response rates across time) and country of birth (non-Swedish-born being either underrepresented in two cohorts by 2 to 3% units or showing declining response rates across time).

During the first year after graduation nearly all participants had qualified as nurses and had later also held a nursing position. The most common reason for not working was due to maternity leave. In the EX2002 and EX2004 cohorts about 10% intended to leave the profession one year after graduation, and in the EX2006 cohort the figure was almost twice as high. In all cohorts, intention to leave the profession was more common among young nurses. In EX2006, twice as many nurses reported having taken active measures to seek positions outside the profession as compared with the other two cohorts. In the first cohort (EX2002) nearly every fifth registered nurse moved on to further higher educational training within the health professions, e.g. midwifery or specialist nursing of some kind. Furthermore in this cohort, about 2% of the participants had left the nursing profession five years after graduating.

### Nursing education and the labour market

The high rates of graduates in the LANE study that receive their nursing qualification, and eventually also hold a nursing position, reflect current trends in Sweden. In the first place, the dropout rate from nursing programmes is generally lower than from other undergraduate programmes in Sweden [56], and between 1988 and 2002 the attrition rate ranged between 8% and 17%. The limited number of international studies that investigate attrition from undergraduate programmes show slightly higher attrition rates, ranging from 19 to 25% [8,17,57]. One factor that may have influenced why nursing graduates completed their education could be the high rates of satisfaction found in a recent survey from the Swedish National Agency for Higher Education. Among those who graduated in 2004, 31% were very satisfied, and 59% were fairly satisfied with their education [58]. In Gaynor and co-workers' review on student retention, attrition was mainly related to incongruence between student expectations and reality [8]. Other results from the LANE EX2002 cohort recently published, showed that the graduates considered their education successful with respect to their development of both general and professional skills [59]. Interestingly, the extent to which former students felt well prepared varied substantially between the 24 Swedish universities. Further studies will be performed to address whether these differences within institutes of higher education persist into working life, and if they do, whether they affect professional performance in practice.

The high rate of new graduates that had held a nursing position at some point since graduation, as well as the fact that the main reason for not working one year after graduation was maternity leave, was in accordance with national statistics on entrance to the labour market [58]. Data from the EX2002 cohort revealed that almost all respondents had held a nursing position at some time during the first five years after graduating. This is in line with the national cohort study from England, where nearly all respondents worked as nurses during the first years after their graduation [60].

Due to the increased number of students that have been accepted for undergraduate nursing programmes, there is currently a balance in supply and demand of nurses in Sweden. However, the present stability in the registered nursing workforce may change and turn into a shortage if issues regarding working conditions and intention to leave the profession are not addressed [61-64]. In both 2002 and 2004, 10% of nurses were thinking of leaving the profession one year after graduating; however, intention to leave was twice as common among 2006 graduates (20%). Since these proportions were also reflected in reports of actively taking measures to leave, this might suggest an increasing problem ahead, especially since intention to leave the profession is consistently higher in the youngest groups. A decrease, especially in younger nurses, was also found in the US [16] and Canada [15]. The differences among the three LANE cohorts in levels of intention to leave may also reflect cohort or period effects. Both the differences in demographic compositions, as well as trends in the labour market for newcomers, may influence the higher proportions of nurses in the EX2006 cohort wanting to leave the profession one year after graduation.

## Method discussion

Initially the LANE study comprised two cohorts, i.e. EX2002 and EX2004, in order to disclose potential cohort effects on the results. The basis for choosing year 2002 for initiation of the study was twofold. First, there was an urgent need to start the investigation in 2002 due to the increasing frequency of stress [25,26] and long-term sick leave [27] among nurses. Owing to the prospective longitudinal study design, the participants had to be still in education, and therefore it was appropriate to carry out the first data collection for cohort EX2002 in the last year of their education. At the same time, a more detailed investigation of the study period was mandatory. In order to secure data from all three years of education, cohort EX2004 was also formed in year 2002, but here study participants were in their first year of education. Second, the concurrent changes within the educational system, where higher nursing education increased in size, dimension [65], started in 2003, i.e. between the formation of the two cohorts. In this way, changes in learning conditions can be studied in relation to possible short- and long-term effects on nurses' educational, clinical and health outcomes. Finally, the last cohort, i.e. EX2006, was formed later, with the same interval as the first two, in order to serve as yet another control group, both for the first two LANE cohorts and for a similar study on teachers (The PATH study; Prospective Analysis of Teachers' Health). Both EX2006 and the PATH cohort were in their final year of education in 2006.

The time frame selected in the LANE study for investigation of common career pathways, life transitions and change in psychological health, covers a period from studies to practice. This is a strength when evaluating the possible influence of educational, working and individual factors on health development and the retention of graduates in the profession. The number of time points in each cohort was chosen to maximize coverage before and after the adjustment and transitional process from student to graduate nurse. Described from a transitional perspective, our assessments were made at both ends of the transition to professional practice, i.e., before and after "the confusing nowhere of in-betweenness", where change is inevitable and the individual will need to develop approaches for dealing with it in one way or another [66]. The transition was understood to affect individuals differently, but to affect participants physically, mentally and socially [66] during the initial year of transition [67]. Recently, Duchscher showed that after about a year, nursing graduates entering professional practice felt accommodated, and that this first year involved both personal and professional qualities. Also, as stated earlier, studies on professional turnover show that some new nurses leave nursing within a few years of entering the profession [15,8,17]. Due to the problem of time lags being too short, involving a risk of missing the phenomena entirely, a longer time lag was chosen, since the risk is then merely a matter of underestimation [68]. Hence, after one year most new graduates were assumed to have got beyond this initial phase, and since Zapf and colleagues (1996) recommend time lags that are too long rather than too short, one year seemed a reasonable approximation of optimal measurement. The one-year interval was chosen based on the idea that participants should have somewhat adapted to their new situation as registered nurses, and that equal time lags were recommended [68]. With regard to longitudinal assessments of career pathways, life transitions and health changes during the first years of working life, it is an advantage to have repeated assessments at approximately the same time every year, due to seasonal differences and changes. Specifically, all assessments after graduation have been performed between February and April each year. This time also coincides with the collection of official statistics (by Statistics Sweden) regarding higher education and employment rates of new graduates [58]. Thus, LANE data can efficiently be compared with official population statistics. In sum, the selection of time points for data collection was mainly based on striking a balance between two issues: 1) the aim to cover a wide variety of research areas (e.g. individual conditions, educational structures, health trends, mental ill health and well-being, and contextual factors in healthcare), and 2) the provision of maximum opportunity to compare the cohorts and control for above-mentioned cohort effects.

The LANE study resembles other concurrent cohort studies on new graduates' transition to practice, such as the Australian e-cohort study (including 540 nursing students) [69], in that it focuses both on retention and employment patterns, as well as on prevalence of musculoskeletal symptoms and work-based injuries. However, unlike the e-cohort study, the main focus of the LANE study is on mental disorders and psychological well-being. Similar to a national cohort study in England (n = 2784), the sampling frame included students representing all nursing programmes in the country [60,70]. In addition, these two studies both follow new graduates for three years after graduation, but differ in that LANE also includes data collections during the respondents' years in higher education.

### Strengths and limitations

When data collection and analyses are complete, the LANE study will add unique knowledge, since surprisingly few studies have actually collected information both during education and after entering nursing practice [71,72,7]. Duchscher emphasises that, although several studies now focus on investigating the effect of different orientation programmes on new graduates' experiences of moving into a professional nursing practice role, she has identified a lack of studies exploring pre-graduate transition preparation. Students' lack of familiarity with what awaits them after graduation, i.e. "the element of surprise", may have a negative effect on new graduates' professional role adaptation [71].

Even if the wave response rates are generally high, they decrease over time. The possible selection bias introduced by this phenomenon must be carefully scrutinized in relation to each particular research question. Specifically, we will compare and contrast attrition due to leaving the profession, embarking on specialist training or being on maternity leave. Although our findings were not constant over the three cohorts, the analyses in this paper generally indicated that gender and country of birth influenced participation and retention. Firstly, the lower male participation (2% units) in the EX2006 cohort, as compared with the population is a phenomenon that has been reported earlier in similar studies. For instance, in the European NEXT (Nurses' Early Exit) study, there was a smaller proportion of men in the study sample, as compared with the percentage in the national workforce, in eight out of eleven countries [14]. Similarly, 9% of the Australian nurse workforce was male, whereas only 6% participated in the study by Turner and co-workers [69]. In the EX2002 cohort, men's response rate instead declined over time. This also seems to be a common trend in comparable studies, where fewer men than women responded to follow-up questionnaires [73-76]. One possible explanation for the smaller number of male respondents could be linked to findings showing that men differ from women in that they: less often enter nursing as a first choice [77], less often complete their education [56,78], have a more critical view towards nursing education [58] and are more inclined to leave the profession [79,80]. As a result, men can be assumed to be less interested in participating in a study directly addressing nursing issues.

The number of immigrants consenting to participate in the EX2002 (6%) and the EX2006 (9%) cohorts was an underrepresentation as compared with the wider population (9% and 11% respectively). In this study, language difficulties cannot be ruled out as a reason for immigrant non-participation; however, this seems to be a less probable cause, since all participants were recruited from higher education. It is difficult to know whether cultural differences can explain the fact that immigrants were less likely to consent to participate, and whether respondents were more integrated into Swedish society than non-participants. In the EX2004 cohort, on the other hand, where the non-Swedish-born subgroup had lower response rates across time, this may be related to higher mobility and a tendency to move out of the country.

When understanding and interpreting different outcome areas in the LANE study, it is important to remember that the population of students at two sites of learning could not be defined prior to the study, and that students from these universities had to personally take the initiative to become part of the sampling frame. Although this selection may not be a major problem (at least not for the EX2002 cohort), exclusion of the consenting students from these two universities will be optional when comparing educational outcomes across universities with regard to data from the EX2002 and EX2004 cohorts. This reservation does not concern the EX2006 cohort, where the total population of students attending the last semester of the nursing programme in the autumn of 2006 could be defined in advance and included in the sampling frame. This limitation will be controlled for when contrasting educational data among the universities.

The main weakness of the study is that data are only collected through self-reports; thus, health data are not clinically validated. However, when data collection closes in 2010, additional data from national registers available for research will be used to form parallel cohorts; data on graduation, employment, maternity leave and sick leave will then be extracted and compared with LANE data.

## Conclusions

The LANE study will provide a unique opportunity to answer a variety of research questions about the transitional development of health and issues in early professional development. The establishment of longitudinal cohorts with high response rates and low attrition over time is necessary in order to estimate prevalence, incidence and factors associated with career pathways, life transitions during the first years of working life, as well as development of depression, burnout, subjective well-being and job engagement in the transition between undergraduate studies and practice.

The use of multiple cohorts is one of the strengths of the LANE study, and this is important in order to disentangle possible cohort and period effects from the developmental trends that are one main focus. An additional strength is that all three cohorts comprise students from all 26 universities offering undergraduate nursing programmes within the higher education system in Sweden. Also, the relatively high response rate when the cohorts were formed, as well as subsequent high response rates and data suggesting high professional retention, imply a potential for a thorough analysis of professional practice and occupational health.

## Competing interests

The authors declare that they have no competing interests.

## Authors' contributions

AR contributed to the design of the study, participated in the acquisition and analysis of data, and drafted the manuscript. MOP contributed to the design of the study, participated in the acquisition of data, and in revising the manuscript. LW contributed to the design of the study, and participated in revising the manuscript. PG contributed to the design of the study, analysed the data and drafted an original version of this manuscript. All authors have read and approved the final manuscript.
